# Effects of Exercise Training on Neurotrophic Factors and Subsequent Neuroprotection in Persons with Multiple Sclerosis—A Systematic Review and Meta-Analysis

**DOI:** 10.3390/brainsci11111499

**Published:** 2021-11-12

**Authors:** Mette D. Diechmann, Evan Campbell, Elaine Coulter, Lorna Paul, Ulrik Dalgas, Lars G. Hvid

**Affiliations:** 1 Exercise Biology, Department of Public Health, Aarhus University, DK-8000 Aarhus C, Denmark; mdd@ph.au.dk (M.D.D.); dalgas@ph.au.dk (U.D.); 2 Healthcare Improvement Scotland, Glasgow G1 2NP, Scotland, UK; evan.campbell@nhs.scot; 3 Department of Physiotherapy and Paramedicine, School of Health and Life Sciences, Glasgow Caledonian University, Glasgow G4 0BA, Scotland, UK; Elaine.Coulter@gcu.ac.uk (E.C.); Lorna.Paul@gcu.ac.uk (L.P.)

**Keywords:** multiple sclerosis, neurotrophic factor, neuroprotection, exercise training, brain-derived neurotrophic factor, meta-analysis

## Abstract

Background: Evidence indicates that exercise holds the potential to counteract neurodegeneration experienced by persons with multiple sclerosis (pwMS), which is in part believed to be mediated through increases in neurotrophic factors. There is a need to summarize the existing evidence on exercise-induced effects on neurotrophic factors alongside neuroprotection in pwMS. Aim: To (1) systematically review the evidence on acute (one session) and/or chronic (several sessions) exercise-induced changes in neurotrophic factors in pwMS and (2) investigate the potential translational link between exercise-induced changes in neurotrophic factors and neuroprotection. Methods: Five databases (Medline, Scopus, Web of Science, Embase, Sport Discus) were searched for randomized controlled trials (RCT) examining the effects of exercise (all modalities included) on neurotrophic factors as well as measures of neuroprotection if reported. The quality of the study designs and the exercise interventions were assessed by use of the validated tool TESTEX. Results: From N = 337 identified studies, N = 14 RCTs were included. While only N = 2 of the identified studies reported on the acute changes in neurotrophic factors, all N = 14 RCTs reported on the chronic effects, with N = 9 studies revealing between-group differences in favor of exercise. This was most prominent for brain-derived neurotrophic factor (BDNF), with between-group differences in favor of exercise being observed in N = 6 out of N = 12 studies. Meta-analyses were applicable for three out of 10 different identified neurotrophic factors and revealed that exercise can improve the chronic levels of BDNF (delta changes; N = 9, ES = 0.78 (0.27; 1.28), *p* = 0.003, heterogeneity between studies) and potentially also ciliary neurotrophic factor (CNTF) (N = 3, ES = 0.24 (−0.07; 0.54), *p* = 0.13, no heterogeneity between studies) but not nerve growth factor (NGF) (N = 4, ES = 0.28 (−0.55; 1.11), *p* = 0.51, heterogeneity between studies). Indicators of neuroprotection (e.g., with direct measures of brain structure assessed by MRI) were assessed in N = 3 of the identified studies only, with N = 2 partly supporting and thus indicating a potential translational link between increases in neurotrophic factors and neuroprotection. Conclusion: The present study reveals that exercise can elicit improvements in chronic levels of BDNF in pwMS, whereas the effects of exercise on chronic levels of other neurotrophic factors and on acute levels of neurotrophic factors in general, along with a potential translational link (i.e., with exercise-induced improvements in neurotropic factors being associated with or even mediating neuroprotection), are sparse and inconclusive. There is a need for more high-quality studies that assess neurotrophic factors (applying comparable methods of blood handling and analysis) concomitantly with neuroprotective outcome measures. Review Registration: PROSPERO (ID: CRD42020177353).

## 1. Introduction

Multiple sclerosis (MS) is a chronic inflammatory and neurodegenerative disease (involving damage of myelin, oligodendrocytes, and axons) [1,2], affecting both the structure and function of the central nervous system (CNS) [3,4]. The damage inflicted by MS leads to the development of a variety of symptoms, such as physical impairments (e.g., walking capacity), cognitive impairments, and fatigue, ultimately affecting quality of life [5].

Interestingly, physical exercise has been shown to partly remedy these outlined symptoms in persons with MS (pwMS), particularly when involving aerobic training and resistance training (individually or in combination), which are the two most commonly examined and applied exercise modalities [6,7,8]. Moreover, a theory that has emerged and matured over the past couple of decades is that exercise also holds neuroprotective effects that may ultimately impact disease progression by counteracting further neurodegeneration [8]. In this review, neuroprotection comprises the partial-to-complete preservation or restoration of neuronal structure and/or function [9]. Preliminary evidence exists to suggest that exercise can elicit neuroprotection in pwMS. Specifically, the preservation/restoration of brain tissue/structure (e.g., cortical thickness, brain volume) as well as brain function (e.g., cortical excitability) have been observed following 10–24 weeks of exercise, including both resistance training [10] and aerobic training [11,12,13]. Nevertheless, it is worth noting that other exercise studies have failed to observe any noticeable neuroprotective effects [14,15].

Such exercise-induced neuroprotective effects are believed to be mediated in part through increased levels of neurotrophic factors [16,17], which are signaling proteins that are secreted to regulate the survival, morphology, and physiology of neurons [18]. Using MS animal models, numerous studies have provided strong evidence confirming that aerobic training and resistance training can increase the acute and chronic expression of neurotrophic factors both systemically as well as locally in the brain [16,17,19,20,21,22]. While a variety of neurotrophic factors have been examined (e.g., ciliary neurotrophic factor (CNTF), neurotrophin3/4/5 (NT3/4/5), glial cell-derived neurotrophic factor (GDNF), insulin-like growth factor 1 (IGF-1)), the majority of studies have targeted brain-derived-neurotrophic-factor (BDNF) and nerve growth factor (NGF). The secretion of BDNF and NGF (along with other neurotrophic factors) are believed to occur within the CNS and the PNS due to neuronal activity [23,24,25] or following the release of different signaling factors from other tissues/organs into the bloodstream (e.g., myokines from exercising skeletal muscles) [26,27,28].

A number of reviews focusing exclusively on pwMS have previously addressed the effects of exercise and neurotrophic factors [5,29], yet none of them have provided a quantitative summary of the existing evidence. Moreover, several exercise studies reporting data on neurotrophic factors (BDNF, NT4, NGF, etc.) in pwMS have been published within the past 3–4 years. Lastly, no studies emphasize whether a direct translational link exists between exercise-induced changes in neurotrophic factors and neuroprotection. Therefore, an updated systematic review combined with meta-analysis is warranted. The objectives of the present systematic review and meta-analysis were to (1) review the evidence on exercise-induced changes in neurotrophic factors in pwMS and (2) explore whether any of the identified studies concomitantly report on outcomes related to neuroprotection (e.g., preservation/restoration of brain structure/function).

## 2. Methods

This study was approved and registered in PROSPERO with registration number CRD42020177353 on 14 July 2020.

### 2.1. Literature Search

The following five electronic databases were searched on 15 of December 2020 and updated on 7 of July 2021: MEDLINE, Embase, Web of Science, Scopus, and Sports Discus. Systematic searches were undertaken combining free text and subject headings. Additionally, a manual search was performed from references in the articles that were included from the systematic search. Further information on the search strategy is displayed in the Supplementary Table.

### 2.2. Selection Criteria 

All included studies had to be either a randomized controlled trial (RCT) or non-randomized controlled trial (CT) with participants having a confirmed diagnosis of MS according to established criteria available at the time of study conduction [30,31]. Furthermore, the identified articles had to include exercise training of any kind (i.e., a structured bodily movement aiming to improve or maintain physical fitness, according to the definition by Caspersen et al. [32]) and report data on at least one neurotrophic factor. Studies were excluded in cases of no available full-text or if the English version was unavailable.

### 2.3. Screening Process

After removal of duplicates, two reviewers (L.G.H. and M.D.D.) undertook the process of screening the articles retrieved from the systematic search and additional searches. The initial screening was based on examination of title and abstracts. Final inclusion was made after a thorough reading of full texts to check whether the articles fulfilled the inclusion criteria. In cases of ambiguities concerning inclusion, a third reviewer (U.D.) was consulted in order to obtain consensus.

### 2.4. Quality Assessment

All included studies were evaluated with the use of the validated Tool for the assessment of Study quality and reporting in Exercise (TESTEX) (Table 1). Each study was graded and given 0–15 points based on relevant parameters assessing study quality and study reporting. TESTEX has, by the developers, been shown to be a reliable tool that facilitates the review of exercise/training trials [33]. While no validated cut-off scores exist for the TESTEX, we used the median score to categorize the identified studies as either higher quality (TESTEX score above the median score) or lower quality (TESTEX score at or below the median score). Two reviewers (M.D.D. and L.G.H.) assessed the included studies independently, which was followed by a consensus scoring (based on a joint review of the individual scores and settlement of any discrepancies).

### 2.5. Data Extraction 

Following screening and quality assessment, descriptive data were extracted from the identified studies (if presented) including MS type, number of participants, Expanded Disability Status Scale (EDSS) score, age, time since diagnosis (TSD), exercise training modality, duration (total number of sessions as well as number of weeks), intensity, frequency (sessions/week). Data extraction of neurotrophic factors and physiological adaptations to exercise training were collected as mean change ± standard deviation (SD) (normally distributed data) or as median ± interquartile range (IQR) (non-normally distributed data). When only figures/illustrations were used to display the relevant data, it was decoded with the online software Web Plot Digitizer [34]. Extracted data of neurotrophic factors are reported as ng·mL^−1^.

### 2.6. Meta-Analysis

A meta-analysis was performed on neurotrophic factors across exercise training modalities, when at least two studies reported relevant data. Meta-analysis was performed according to the guidelines of the Cochrane Handbook Chapter 9: Analyzing data and undertaking meta-analyses [35], using delta (pre–post) mean values ± standard deviation (SD). In case delta SD was not reported, this was calculated based on pre and post SD values: SD_Δ_ = ^2^√(SD_Pre_^2^ + SD_Post_^2^ + (2 × correlation × SD_Pre_ × SD_Post_)). Regarding the correlation value, since none of the identified studies reported a pre and post-test correlation, we assumed a conservative correlation of 0.7. When studies reported data on more than one exercise training intervention group, the data from the exercise training groups were pooled into a single group as recommended [35]. Random effects meta-analyses were carried out using Review Manager (version 5.4, The Cochrane Collaboration). Intervention effect sizes (ES) (between-group differences) for the neurotrophic factor outcomes (pre–post (delta) values) were calculated using Hedges’ g statistic along with 95% confidence intervals (CIs) around the estimated effects size. ES were interpreted based on summarized empirical data derived from 99 meta-analyses examining the effects of rehabilitation treatment: small = 0.14, medium = 0.31, and large = 0.61 [36]. Statistical heterogeneity was quantified using Higgins’ I^2^ statistic and was interpreted as follows: heterogeneity: >50%, no or limited heterogeneity: <50% [37].

## 3. Results

### 3.1. Screening Process and Study Selection

**Figure 1:**
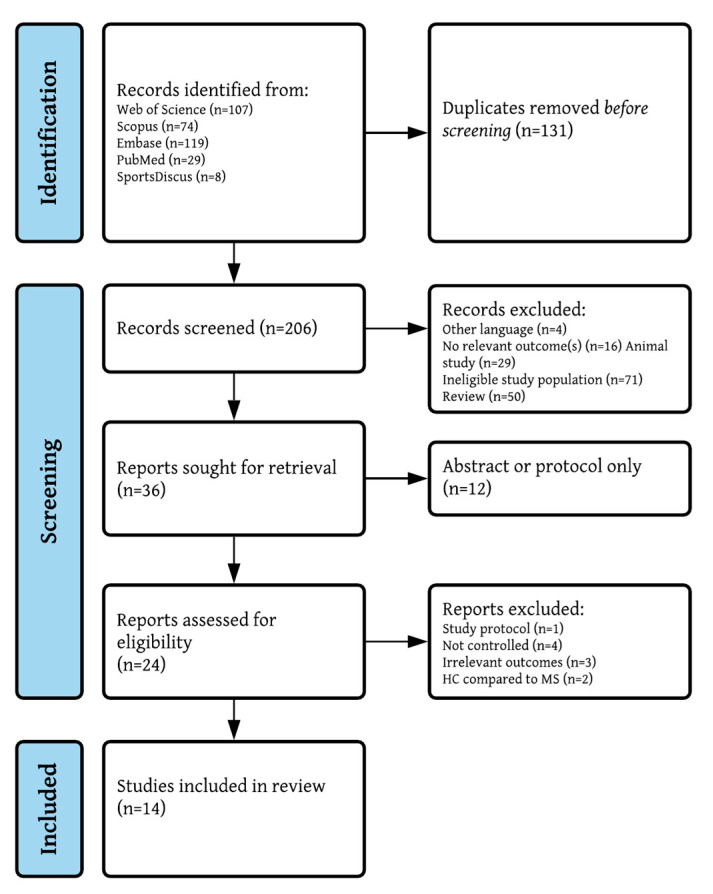
PRISMA flowchart.

Figure 1 presents a PRISMA flowchart of the study selection and screening process. Of the N = 337 studies identified from databases, N = 14 studies fulfilled the inclusion criteria [6,13,38,39,40,41,42,43,44,45,46,47,48,49]. No additional studies were found through reference lists of the included studies.

### 3.2. Study Quality

Based on the TESTEX scores, the identified N = 14 studies obtained a median score of 8.5 (range 3–12) (Table 1), with N = 7 studies being considered higher quality (TESTEX scores > median) [13,39,40,44,45,47,48] and N = 7 studies being considered lower quality (TESTEX scores ≤ median) [6,38,41,42,43,46,49]. Two of the criteria items were not met in any of the studies: “intention-to-treat analyses” and “activity monitoring in control group”. In addition, only N = 4 studies were specific on the randomization method being used [40,41,44,45], and only N = 7 studies had groups (i.e., exercise vs. control) that were comparable at baseline [6,13,38,44,45,47,49]. As for the training interventions, the relative intensity was held constant as progression was applied in N = 11 studies [13,38,39,40,41,42,44,45,46,47,48], and N = 13 studies provided specific details on the intervention parameters (training modality, session frequency and duration, intensity) [6,13,38,39,40,41,42,43,44,45,46,47,48].

### 3.3. Participants

The majority of pwMS participants were women (366 out of 473 participants, 77%), with a mean age of 37.4 years and EDSS scores ranging from 0 to 6.5 (i.e., from no disability to ability to walk with bilateral assistive devices [50]) (median EDSS = 2.6) (Table 2). Only one study [39] recruited pwMS with EDSS scores above 6.5 corresponding to high disability, with 19 out of 89 participants having EDSS scores between 6.5 and 8.0 (no further details were provided). Most participants had relapse–remitting MS, as only three studies [6,40,42] mentioned the inclusion of participants having primary or secondary progressive MS. TSD ranged from 4 to 16 years (mean TSD = 9.7 years) based on the seven studies that reported TSD.

### 3.4. Exercise Interventions 

The modalities of the identified studies were resistance training (RT, N = 1) [47], Pilates (N = 1) [41], aerobic training (AT, N = 5) [6,13,40,48,49], or a mix of training regimes (mixed, N = 7) [38,39,42,43,44,45,46]. RT consisted of four lower and two upper body exercises, carried out using machines [47]. AT consisted of arm ergometry, cycle ergometry, aquatic exercise, aerobic, or rowing [6,13,42,48,49]. Mixed consisted of either a combination of AT and RT, a combination of AT, RT, and stretch + balance + Pilates, or a combination of AT and massage [38,39,42,43,44,45,46]. One study [46] had three groups: AT, RT, and a control group. Table 2 provides detailed information on the intervention of each study. Apart from divergent exercise modalities, the specification of each intervention also differed in sessions per week (two to five sessions per week), intervention duration (6–24 weeks, 16–60 sessions), session duration (15–100 min), and session intensity (deemed as moderate-to-high intensity for both RT and AT).

### 3.5. Physiological and Functional Outcome Measures

The included studies used a variety of physiological outcome measures (Table 2), including muscle strength of the lower (e.g., knee extension and knee flexion) and upper extremities (e.g., hand grip) as well as aerobic capacity (i.e., VO_2max_). Furthermore, they included a variety of functional outcome measures (e.g., walking distance or speed, balance, disability status). N = 3 studies [41,46,49] did not report on any physiological or functional outcome measures. Overall, there were positive findings, as N = 10 studies reported between-group differences in favor of exercise for at least one of the physiological or functional outcome measures [13,38,39,40,42,43,44,45,47,48]. These between-group differences were predominantly due to significant within-group improvements in the intervention groups paralleled by no changes in the control groups. Of note, one study did not observe any between-group differences in their physiological outcomes [6]. 

### 3.6. Acute Effects of Exercise on Neurotrophic Factors

As displayed in Table 2, only N = 2 studies examined the acute effects of exercise on neurotrophic factors. Joergensen et al. [47] assessed the acute effects of RT on BDNF (0, 15, 45, 75, 120 min after exercise) but found no within- or between-group differences prior to the 24-week RT intervention or any within-group differences in the exercise group following the intervention (between-group differences were not applicable as the study did not assess the control group after the intervention). Rezaee et al. [49] assessed the acute effects of AT on vascular endothelial growth factor (VEGF) (immediately after exercise) and found within- as well as between-group differences in favor of exercise, both prior to and following the 6-week AT intervention (1st and 18th session, respectively). Nevertheless, the magnitude of acute effects was comparable across these two sessions.

### 3.7. Chronic Effects on Neurotrophic Factors

**Figure 2:**
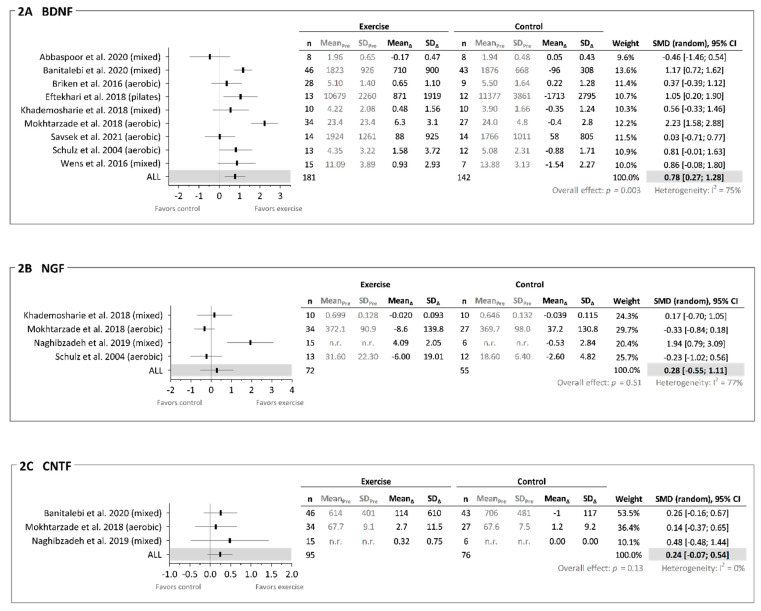
Meta-analysis of the effects of exercise on chronic levels of neurotrophic factors. (**A**): Chronic levels of BDNF (brain-derived neurotrophic factor). (**B**): Chronic levels of NGF (nerve growth factor). (**C**): Chronic levels of CNTF (ciliary neurotrophic factor). Units of neurotrophic factors are displayed as ng·mL^−1^. See Table 1 and 2 for further study details.

All N = 14 included studies assessed and reported at least one neurotrophic factor before and after an exercise intervention, as displayed in Table 2 and in Figure 2A–C (BDNF, NGF, and CNTF only). Overall, the effects of exercise on neurotrophic factors appeared sparse and inconclusive, except for the robust improvements observed in BDNF (based on number of studies reporting between-group improvements and on the meta-analysis result; see below). Results from some neurotrophic factors (IGF-1, NT3, NT4, NT4/5, GDNF, PDGF, and VEGF) were assessed in and reported from individual studies only, excluding the possibility of providing an overall robust conclusion including performing meta-analyses. Apart from measuring neurotrophic factors from either blood serum or plasma, the studies varied in their timing and handling of the blood samples. Blood samples were taken from 30 min to 48 h after the last exercise bout, were carried out using different commercially available enzyme-linked immunosorbent assays (ELISA) kits, and involved variations in timing as well as the method of centrifugation (further information in Table 2).

#### 3.7.1. Brain-Derived Neurotrophic Factor (BDNF)

N = 12 studies reported on the effects of exercise on chronic BDNF levels (plasma n = 2, serum n = 10). While N = 4 studies failed to observe between- or within-group differences in BDNF levels [6,38,40,47], N = 2 studies [43,44] observed within-group differences in the exercise groups but no between-group changes. The remaining N = 6 studies [13,39,41,42,45,48] reported between-group differences in favor of exercise, which was due to an improvement in the exercise group only [13,39,48] or due to a decrement in the control group concomitantly with an increment in the exercise group [41,42,45] (exact statistics not mentioned in two of the studies [41,42]).

As N = 2 studies [44,47] reported non-normally distributed data (median (IQR)), these data could not be included in the meta-analysis (of note, these two studies did not observe any between-group changes in chronic BDNF levels). N = 6 studies did not mention whether they checked for data normal distribution [6,13,39,40,43,48], whereas N = 5 studies [38,41,42,45,46] reported the statistical tool they used for normality check (yet not the exact outcome of the tests). N = 1 study reported p-values only but not the exact data [43] and thus also could not be included in the meta-analysis. For the remaining N = 9 studies, meta-analysis across all exercise modalities revealed a large and certain ES when based on pre–post change (delta) BDNF data (N = 9, ES = 0.78 (0.27; 1.28), I^2^ = 75%, *p* = 0.003; Figure 2A) in favor of exercise. Serving as sensitivity analysis, the removal of individual studies (e.g., the visual outliers by Abbaspoor et al., 2020 and Mokhtarzade et al., 2018) did not affect the overall result, i.e., a large and certain ES remained (data not shown).

Across separate exercise modalities, a large yet slightly uncertain ES was observed for aerobic interventions (N = 4, ES = 0.87 (−0.15; 1.90), *p* = 0.10, I^2^ = 87%) as well as for mixed interventions (N = 4, ES = 0.61 (−0.07; 1.30), *p* = 0.08, I^2^ = 67%). The remaining N = 1 was for Pilates (N = 1, ES = 1.05 (0.20; 1.90)). Further meta-analyses were also carried out according to the quality of the identified studies, with a medium-to-large yet slightly uncertain ES observed for lower quality studies (TESTEX score < 8.5; N = 4 studies, ES = 0.53 (−0.08; 1.14), *p* = 0.09, I^2^ = 47%) alongside a large and certain ES for higher-quality studies (TESTEX score > 8.5; N = 5, ES = 0.95 (0.22; 1.69), *p* = 0.01, I^2^ = 83%).

#### 3.7.2. Nerve Growth Factor (NGF)

N = 4 studies reported on the effects of exercise on chronic NGF levels (plasma N = 1, serum N = 3). N = 2 studies [42,48] reported no within- or between-group differences in NGF. N = 1 study [43] observed between-group differences in favor of exercise due to an improvement in the exercise group, and N = 1 study [6] reported a trend for a negative between-group difference in NGF due to a decrement in the exercise group. For these N = 4 studies, meta-analysis across all exercise modalities revealed a medium yet very uncertain ES when based on pre–post change (delta) NGF data (N = 4 studies, ES = 0.28 (−0.55; 1.11), *p* = 0.51, I^2^ = 77%; Figure 2B) in favor of exercise.

Across separate exercise modalities, a medium yet uncertain *negative* ES was observed for aerobic interventions (N = 2 studies, ES = −0.30 (−0.73; 0.12), *p* = 0.16, I^2^ = 0%) in favor of control, along with a large yet very uncertain ES for mixed interventions (N = 2 studies, ES = 1.02 (−0.71; 2.75), *p* = 0.25, I^2^ = 83%) in favor of exercise. Across the quality of the identified studies, a medium-to-large yet very uncertain ES was observed for lower quality studies (TESTEX score < 8.5; N = 3 studies, ES = 0.56 (−0.61; 1.74), *p* = 0.35, I^2^ = 79%) in favor of exercise, with the remaining N = 1 study being a higher quality study (TESTEX score > 8.5; N = 1, ES = −0.33 (−0.84; 0.18)).

#### 3.7.3. Ciliary Neurotrophic Factor (CNTF)

N = 3 studies reported on the effects of exercise on chronic CNTF levels (plasma N = 1, serum N = 2. These studies [39,43,48] reported no within- or between-group differences. For these N = 3 studies, meta-analysis across all exercise modalities revealed a small-to-medium yet slightly uncertain ES (N = 3 studies, ES = 0.24 (−0.07; 0.54), *p* = 0.13, I^2^ = 0%; Figure 2C) in favor of exercise. Further meta-analyses were also carried out, revealing small-to-medium yet slightly uncertain ES of mixed interventions (N = 2, ES = 0.29 (−0.09; 0.67), *p* = 0.14, I^2^ = 0%) (the remaining N = 1 an AT study (N = 1, ES = 0.14 [−0.37; 0.65])) and of higher quality studies (N = 2, ES = 0.21 (−0.11; 0.53), *p* = 0.20, I^2^ = 0%) (the remaining N = 1 study a lower quality study (TESTEX score < 8.5; N = 1, ES = 0.48 [−0.48; 1.44])).

#### 3.7.4. Insulin-Like Growth Factor-1 (IGF-1)

N = 1 study reported on the effects of exercise on chronic IGF-1 levels (serum N = 1), with the mixed intervention revealing within- and between-group improvements [38].

#### 3.7.5. Neurotrophin 3 (NT3), 4 (NT4), and 4/5 (NT 4/5)

N = 2 studies reported on the effects of exercise on chronic neurotrophin levels (plasma N = 1, serum N = 1). N = 1 study reported between-group differences for both NT3 and NT4/5 after a mixed intervention in favor of exercise [39], whereas another N = 1 study reported within- and between-group differences for NT4 after both RT and AT (aquatic) (separately compared to control) in favor of exercise [46] achieved due to increases in the intervention group.

#### 3.7.6. Glial Cell-Derived Neurotrophic Factor (GDNF)

N = 1 study reported on the effects of exercise on chronic GDNF levels (serum N = 1), with the mixed intervention revealing no within- or between-group differences [39].

#### 3.7.7. Platelet-Derived Growth Factor (PDGF)

N = 1 study reported on the effects of exercise on chronic PDGF levels (serum N = 1), with the AT intervention revealing within- and between-group differences in favor of exercise [48] achieved due to increases in the intervention group.

#### 3.7.8. Vascular Endothelial Growth Factor (VEGF)

N = 1 study reported on the effects of exercise on chronic VEGF levels (serum N = 1) with the AT intervention revealing no within- and between-group differences [49].

### 3.8. Neuroprotective Effects of Exercise

As previously stated, studies reporting on the neuroprotective effects of exercise may provide a translational link and insight into the role of exercise-induced changes in neurotrophic factors. However, only N = 3 studies reported on outcomes related to neuroprotection. Magnetic resonance imaging (MRI) outcomes were assessed in the same study population as included in Joergensen et al. [47] yet reported separately by Kjolhede et al. [10]. Following 24 weeks of high-intensity RT, between-group differences (preservation) were observed in total brain volume (trend only) and in cortical thickness (four out of 74 regions), whereas the T2 lesion volume and count remained unaffected by the RT intervention [10]. Of note, no between-group differences in chronic plasma BDNF levels were observed (non-normally distributed data, reported as median (IQR); thus, they were not included in the meta-analysis) [47]. MRI outcomes were also assessed by Savsek et al. [13]; following 12 weeks of AT, they observed between-group differences in favor of exercise for the brain volume of some substructures (decrement in two of 15 substructures, increment in one (parahippocampal gyrus) of 15 substructures; preservation in two of 15 substructures) along with a between-group decrement in active lesion volume and count, whereas the total brain volume, gray matter brain volume, T2 lesion volume and count, and cortical lesion volume and count were unaffected by the AT intervention. This was paralleled by between-group increases in chronic serum BDNF levels (trend only). Mokhtarzade et al. [48] used blood–brain-barrier permeability markers (S100 calcium-binding protein B (S100b) and neuron-specific enolase (NSE)) to understand neuroprotection from exercise, i.e., whether blood–brain-barrier structural damage could be attenuated and/or counteracted. Following 8 weeks of AT, NSE remained unaffected, whereas a between-group decrement in S100b was observed (normal-weight pwMS only). This was paralleled by between-group increases in chronic serum PDGF and BDNF levels (normal-weight pwMS only), whereas chronic serum NGF and CNTF levels remained unaffected. None of the studies outlined above ran any analysis of association (e.g., simple correlation) between exercise-induced changes in neurotrophic factors and their neuroprotection outcomes.

## 4. Discussion

To the best of our knowledge, the present systematic review and meta-analysis is the first to quantitatively summarize the existing evidence on the effects of exercise training on neurotrophic factors alongside any subsequent neuroprotection in pwMS (i.e., providing ‘proof of concept’). We identified N = 14 eligible studies (all RCTs, involving n = 473 pwMS) that were heterogeneous in terms of exercise modalities (aerobic N = 5, resistance N = 1, Pilates N = 1, mixed N = 7) and intervention duration (8–24 weeks involving 16–60 sessions), study quality and reporting (TESTEX score range 3–12 (maximal possible score is 15), median score 8.5), as well as the chosen outcome measures. Exercise training elicited positive adaptations in one or more physiological and/or functional outcome measures (e.g., muscle strength, aerobic capacity, walking capacity) in N = 10 studies (N = 1 study failed to observe any adaptations), whereas N = 3 studies did not report on any of these outcome measures. More importantly, exercise elicited positive adaptations in acute levels of neurotrophic factors in N = 1 study (N = 1 study failed to observe any adaptations) and in chronic levels of neurotrophic factors in N = 10 studies (N = 4 study failed to observe any adaptations). The latter was most prominent for BDNF, with N = 6 out of N = 12 studies reporting between-group differences in favor of exercise. Moreover, random effects meta-analyses revealed that exercise across different modalities can improve chronic levels of BDNF (N = 9, large ES certain CI estimate) and potentially also of CNTF (N = 3, small-to-medium ES slightly uncertain CI estimate) but not NGF (N = 4, medium ES very uncertain CI estimate). Lastly, N = 3 studies reported data in relation to neuroprotection (two with direct measures of brain structure assessed by MRI), with two of these partly supporting a translational link between increases in neurotrophic factors and neuroprotection. In summary, the present findings support that exercise elicit a positive effect on chronic levels of BDNF in pwMS, whereas the evidence supporting any effects of exercise on chronic levels of other neurotrophic factors and on acute levels of neurotrophic factors in general, along with a potential translational link (i.e., with exercise-induced improvements in neurotropic factors being associated with (or even mediating) neuroprotection), are sparse and inconclusive.

### 4.1. Acute and Chronic Neurotrophic Response to Exercise

While acute and chronic levels of neurotrophic factors have generally been argued to increase following exercise (BDNF in particular) [51,52,53,54,55,56,57,58], the results from this systematic review and meta-analysis involving pwMS temper such statements. One main limitation is of course the scarcity of studies examining acute and chronic levels of neurotrophic factors in pwMS, with the only exception being the chronic levels of BDNF examined in N = 12 out of the N = 14 identified studies (with N = 6 studies reporting improvements in favor of exercise). The meta-analysis revealed a large and certain ES across all studies examining the chronic levels of BDNF (ES = 0.78, N = 9), which is an observation apparently driven by the studies being categorized as higher-quality studies (ES = 0.95, N = 5). Of note, the two studies that reported non-normally distributed data and were left out of the meta-analysis were both higher-quality studies, with none of them reporting between-group changes in chronic BDNF levels in favor of exercise [44,47]. This must be kept in mind when interpreting these meta-analysis findings.

### 4.2. Exercise Modality

The chosen exercise modality may be essential to whether exercise elicits increases in neurotrophic factors or not. The underlying mechanisms by which cellular changes (neurogenesis, synaptogenesis, angiogenesis) and subsequent structural and/or functional CNS/brain changes can occur, such as improved efferent neural drive and brain volume [59], are of course complex, yet they are proposed to be of both central and peripheral origin. In the former, direct effects occur locally in the brain, with increased neuronal activity leading to increased levels of BDNF and other neurotrophic factors [25]. In the latter, indirect effects occur, with the release of myokines (e.g., irisin) from exercising skeletal muscles into the blood that are subsequently transported to and entering the CNS/brain, leading to increased levels of BDNF and other neurotrophic factors [27,28]. The findings of the present study do not seem to support the superiority of one exercise modality over another in pwMS. This was mainly due to the scarcity of studies across most neurotrophic factors, yet we did observe a numerically greater ES following aerobic vs. mixed interventions (ES = 0.87 vs. ES = 0.61; chronic levels of BDNF). To evaluate this robustly, direct head-to-head comparison of modalities are needed. Only one of the identified studies assessed this, revealing AT and RT to have the same positive influence on chronic levels of NT4 (increased compared to control) [46]. From a historical perspective, aerobic exercise has generally been highlighted as the most potent exercise modality when it comes to increasing levels of neurotrophic factors [59]. This statement is partly supported by our observations (chronic BDNF levels) but also by summary findings of previous reviews (acute and chronic BDNF levels) taking a broad approach by including studies involving both healthy and/or patient populations [52,52,55]. However, a predominant part of studies included in these reviews (including the present one) have applied aerobic exercise, enabling robust analysis and findings across modalities other than aerobic exercise. A similar observation has been made by another recent systematic review examining the effects of exercise on biomarkers in pwMS [29]. This altogether contrasts with the fact that other exercise modalities are commonly applied in pwMS [8], especially resistance training, that are known to be as potent as aerobic training in eliciting positive effects on physical function (e.g., walking capacity) as well as fatigue [60].

An essential aspect of all exercise modalities relates to the volume, intensity, and duration of the exercise interventions. Despite some inconsistency, studies involving healthy individuals have indicated that the reported positive effects of aerobic training and resistance training on neurotrophic factors follow a dose–response relationship regarding volume, intensity, and duration [51,52,53,54,55,56,57,58]. Some studies have reported comparable effects following moderate and high-intensity aerobic training [56] as well as resistance training [58], yet with a superior effect of longer session duration or greater session volume, respectively. Involving pwMS, Zimmer et al. [61] reported acute BDNF increases following 30 min of continuous moderate-intensity aerobic training and 20 min of high-intensity interval aerobic training (HIIT) that did not differ (as this study had no control group, it was not included in the present review). As the vast majority of studies included in the present review employed a narrow range of duration (8–12 weeks in N = 11 studies), session numbers (2–3 per week in N = 14 studies), and intensity (deemed as moderate-to-high in N = 14 studies), we cannot verify the existence of any dose–response relationship on the data of the present review.

Despite the challenges of evaluating the aspects outlined above, one must bear in mind that exercise is a cornerstone in MS rehabilitation that is almost entirely absent of side effects and with numerous health benefits (e.g., on physical function, cardiorespiratory function, neuromuscular function) [8]. This justifies a continuous strong support of exercise as an adjunct treatment strategy for pwMS.

### 4.3. Translation between Neurotrophic Factors and Neuroprotection

Research in animal models of MS has numerous times shown that an exercise-induced increase in neurotrophic factors is one of the mechanisms involved in eliciting structural/functional neuroprotection [16,17]. Such a translational link has been argued to occur in pwMS also [8,62], yet we were able to identify three studies only, examining the effects of exercise on neurotrophic factors concomitantly with outcomes related to neuroprotection [10,13,47,48]. Kjoelhede/Joergensen et al. did not support such a translational link (24 weeks RT; unaffected chronic BDNF levels, preserved whole brain volume, increased cortical thickness of four regions) [10,47]), whereas the remaining two studies did so in part. Savsek et al. showed that 12 weeks of AT increased chronic BDNF levels, which were furthermore accompanied by increased/preserved brain volume of five substructures and decreased active lesion volume and count [13]. However, the change in chronic BDNF levels was a trend only, and they failed to observe changes in total/gray matter brain volume, 12 substructures of brain volume, as well as in T2/cortical lesion volume and count. Mokhtarzade et al. showed that 8 weeks of AT increased chronic PDGF and BDNF levels, which were furthermore accompanied by decreased S100b (blood–brain-barrier permeability marker) [48]. However, this was observed in normal-weight pwMS only but not in overweight pwMS. In addition, they did not observe any changes in chronic NGF or CNTF levels as well as in NSE (another blood–brain-barrier permeability marker). None of the studies outlined above included analysis of association (e.g., simple correlation) between exercise-induced changes in neurotrophic factors and their neuroprotection outcomes. This would theoretically have provided a better insight into a potential causal relationship. Nevertheless, despite the apparent limitations, these preliminary study findings are intriguing. A general hindrance when interpreting such a translational link relates to the temporal resolution of adaptations in neurotrophic factors vs. neuroprotective outcomes, which is an aspect that we currently know little about. Moreover, our current understanding of exercise-induced neuroprotection in pwMS is sparse and inconclusive [8,15,62], with large, long-term (exercise durations ≥1 year; brain/CNS structure in particular), high-quality studies designed specifically to examine the effects of exercise on brain/CNS structure and function being warranted. As evidenced by the present review, this also applies to the effects of exercise on neurotrophic factors, although our novel findings support that chronic BDNF levels can be increased. Future studies should address these issues to help clarify whether such ‘proof of concept’ translational evidence exists or not. In relation to this, the so far promising use of the blood-based biomarkers neurofilament light (NfL) and glial fibrillary acidic protein (GFAP) that are strongly associated with disease progression and specifically neurodegeneration [63,64] should be kept in mind. Some studies have reported that short-term (3–8 weeks) aerobic training can reduce serum/plasma levels of NfL and GFAP [65,66] in pwMS, whereas a long-term (24 weeks) aerobic training study failed to observe any changes in serum NfL [14]. However, none of these exercise studies carried out concomitant analyses of neurotrophic factors, which is an obvious knowledge gap that should be pursued in future.

### 4.4. Methodological Considerations

Some methodological considerations deserve mentioning. First, it is plausible that blood sampling and handling are major causes of heterogeneity across the identified studies, as evidenced by previous methodological studies. Gejl et al. [67] found significantly higher levels of BDNF in serum compared to plasma and found no correlation between serum and plasma measurements, which might indicate that they are representing two different pools of BDNF. Furthermore, they reported high inter-individual variability in serum measurements of BDNF but only low variability in plasma measurements [67]. Analyzing plasma has been proposed to be advantageous as it gives a more undisturbed picture of the free-floating BDNF in the bloodstream, with initiation of the coagulation processes activating the blood plates which then release BDNF [47]. Nevertheless, this fact was used as a counter-argument by Polacchini et al. stating that such rises in the free-floating BDNF levels are distorting the in vivo state and thus misleading [68]. Additionally, Vrijen et al. argued that plasma levels are a reflection of the momentary BDNF levels, whereas serum levels are a reflection of the long-term BDNF levels [69]. In the present review, studies are primarily measured from serum samples (Table 2). Another aspect concerns the precision, sensitivity, and detection range for the kits used to assess neurotrophic factors. Previous BDNF studies have shown these to vary considerably depending on the kits being used [68,69]. These methodological concerns must be taken into account when interpreting the findings of the present review (e.g., variation in absolute levels is evident from the meta-analysis; see Figure 2A–C and Table 2). As no clear pattern emerged from the present data, further methodological studies are clearly warranted to address these concerns. Second, the assessment of study quality is based on TESTEX, which is a new tool in quality assessment that in addition to study design also takes into account the exercise intervention parameters. As the tool has no cut-off point, we decided to use the terms ‘higher’ or ‘lower’ quality in order to consider study quality when interpreting results from the meta-analysis. Third, our meta-analyses are based on as little as three studies for CNTF (four for NGF) and on studies that are heterogeneous in nature (as previously discussed due to differences in exercise modality, intervention duration, study quality and reporting, and chosen outcome measures). With the exception of chronic BDNF levels, this limits the robustness of our conclusions. Fourth, none of the identified studies reported neurotrophic factors as their primary outcome and may thus likely have been underpowered. Fifth, the findings predominantly apply to relapse–remitting pwMS, as few studies included progressive pwMS [9,43,45]. This is relevant, since circulating levels of neurotrophic factors, BDNF in particular, seem to change according to disability progression [39] and thus likely also MS type. However, Banitalebi et al. [39] did not observe any substantial differences in exercise-induced adaptations in neurotrophic factors across pwMS having low, moderate, and high disability (EDSS ranges 0–4.0, 4.5–6.0, and 6.5–8.0, respectively). These notions must all be taken into account when interpreting the results of the present review.

## 5. Conclusions

Across N = 14 identified RCTs, the present study reveals that exercise training can elicit improvements in chronic levels of BDNF in pwMS, whereas the effects of exercise training on chronic levels of other neurotrophic factors and on acute levels of neurotrophic factors in general, along with a potential translational link (i.e., with exercise-induced improvements in neurotropic factors being associated with or even mediating neuroprotection), are sparse and inconclusive. There is a need for more high-quality exercise training studies that assesses neurotrophic factors (applying comparable methods of blood handling and analysis) concomitantly with neuroprotective outcome measures in pwMs.

## Figures and Tables

**Table 1:** TESTEX study quality assessment.

Study	Study Quality					*Sub-Total*	Study Reporting							*Sub-Total*	Total
	1	2	3	4	5		6	7	8	9	10	11	12		
Abbaspoor et al., 2020 (mixed)	1	0	0	1	1	*3*	0	0	2	1	0	1	1	*5*	**8**
Askari et al., 2017 (aerobic + resistance)	1	0	0	0	0	*1*	0	0	2	1	0	1	1	*5*	**6**
Banitalebi et al., 2020 (mixed)	1	0	1	0	1	*3*	2	0	2	1	0	1	1	*7*	**10**
Briken et al., 2016 (aerobic)	1	1	1	0	0	*3*	2	0	1	1	0	1	1	*6*	**9**
Eftekhari et al., 2018 (Pilates)	1	1	0	0	0	*2*	0	0	1	1	0	1	1	*4*	**6**
Joergensen et al., 2019 (resistance)	1	0	1	1	0	*3*	3	0	2	1	0	1	1	*8*	**11**
Khademosharie et al., 2018 (mixed)	1	0	0	0	0	*1*	0	0	2	1	0	1	1	*5*	**6**
Mokhtarzade et al., 2018 (aerobic)	1	0	1	0	1	*3*	2	0	2	1	0	1	1	*7*	**10**
Naghibzadeh et al., 2019 (mixed)	1	0	0	0	1	*2*	0	0	1	0	0	0	0	*1*	**3**
Ozkul et al., 2018 (mixed)	1	1	1	1	1	*5*	3	0	1	1	0	1	1	*7*	**12**
Rezaee et al., 2020 (aerobic)	1	0	0	1	0	*2*	1	0	2	1	0	0	1	*5*	**7**
Savsek et al., 2021 (aerobic)	1	0	0	1	1	*3*	2	0	2	1	0	1	1	*7*	**10**
Schulz et al., 2004 (aerobic)	1	0	1	1	0	*3*	0	0	2	1	0	0	1	*4*	**7**
Wens et al., 2016 (mixed)	1	1	1	1	0	*4*	3	0	2	1	0	1	1	*8*	**12**
Total (across sub-scores)	14	4	7	7	6		18	0	24	13	0	11	13		**Median** **= 8.5**

Study quality: 1, Eligibility criteria specified; 2, Randomization specified; 3, Allocation concealment; 4, Groups similar at baseline; 5, Blinding of assessors. Study reporting; 6, Outcome measures assessed in 85% of patients; 7, Intention-to-treat analysis; 8, Between-group statistical comparisons reported; 9, Point measures and measures of variability for all reported outcome measures; 10, Activity monitoring in control group; 11, Relative exercise intensity remained constant; 12, Exercise volume and energy expenditure.

**Table 2:** Summary of study results.

Study	MS Participants	Intervention	Type of Training	Neurotrophic Factors Primary Outcome		Physiological and/or Functional Adaptations
	MS type (RR/SP/PP) Number of participants (m/f%) EDSS (mean/median score or range) Age (mean years or range) TSD (mean years)		Duration (weeks) Sessions/week (s/wk) Session duration (min) Intensity	(yes/no/not reported) Methods for Assessing Neurotrophic Factors Neurotrophic Factor(s) Adaptations		Neuroprotective Adaptations (yes/no/not reported)
**Abbaspoor et al., 2020**	RR n = 16 (0/100%) Age: 35.4 EDSS: 3.0 TSD: 10.1	1: MIX 2: Control	8 weeks, 3 s/wk *AT (rhythmic)*: 15–20 min, 55–70% of HR_max_ 3 s/wk *TRX* or *elastic band* or *body weight training*: 1–2 × 8–14 reps, 1 s/wk	Not reported		Grip strength ↑_BG_ Finger pinch strength 2 min walking distance (↑_BG_) Walking speed ↑_BG_ Knee ext strength Not reported
				Methods: Blood samples 48 h before and after intervention. Serum analyzed by ELISA kit (BDNF: *Shanghai Crystal Day Biotech Co, China*; IGF-1: *Mediagnost, Germany*). Centrifugation 3000 g for 10 min. Storage −80 °C.		
				Acute:	Chronic: BDNF IGF-1 ↑_BG_	
**Askari et al., 2017**	MS type not reported n = 30 (0/100%) Age: 35.1 EDSS: not reported TSD: not reported	1: AT 2: RT 3: Control NB: results from AT and RT are pooled into a MIX group.	8 weeks, 3 s/wk *AT (aquatic)*: 30 min, no intensity reported *RT*: 30–45 min, 40–70% of 1RM, machines, 2–3 × 10–14 reps	Not reported		Not reported Not reported
				Methods: Blood samples 48 h before and after intervention. Plasma analyzed with ELISA kit (NT4: *Chongqing Biospes Co, China*). Centrifugation not reported. Storage −70 °C.		
				Acute:	Chronic: NT4 ↑_BG (AT and RT vs control)_	
**Banitalebi et al., 2020**	RR n = 89 (0/100%) Age: not reported EDSS 0–4: n = 45 EDSS 4.5–6.0: n = 25 EDSS 6.5–8.0: n = 19 TSD: Not reported	1: MIX 2: Control	12 weeks, 3 s/wk 100 min (in total) *AT (bike or run)*: 50–70% of HR_max_ *RT (whole body)*: 40–70% of 1RM, 3 × 12 reps *Balance*: static + weight shift, no further details reported *Pilates*: no details reported *Stretching*: to pain threshold, no further details reported	Not reported		Knee ext strength ↑_BG_ VO_2max_ ↑_BG_ Body fat % ↓_BG_ Not reported
				Methods: Blood samples before and after intervention (after overnight fasting). Serum analyzed by ELISA kit (BDNF, NT3, NT4/5, GDNF: *Boster Bio, CA, US; CNTF: Stabiopharm, Singapore*). Centrifugation 500 g for 12 min at 4 °C. Storage −80 °C.		
				Acute:	Chronic: BDNF ↑_BG_ NT3 ↑_BG_ NT4/5 ↑_BG_ GDNF CNTF	
**Briken et al., 2016**	SP/PP (31/11) n = 42 (42.9/57.1%) EDSS: 4.9 Age: 50.0 TSD: 16.3	1: AT (arm ergometry) 2: AT (bicycle) 3: AT (rowing) 4: Control	9 weeks, 2–3 s/wk 15–45 min (in total) *AT*: 120–130% of anaerobic threshold	No (primary outcome: Vo_2max_)		Vo_2max_ ↑_BG (bicycle vs control)_ 6 min walking distance ↑_BG (arm and bicycle vs control)_ Not reported
				Method: Blood samples before and after intervention (at rest). Serum analyzed by ELISA kit (BDNF: *Promega, WI, US*). Centrifugation not reported. Storage −80 °C.		
				Acute:	Chronic: BDNF	
**Eftekhari et al., 2018**	RR (Interferon-β) n = 25 (0/100%) EDSS: 2.0–6.0 Age: 33 TSD: not reported	1: Pilates (mat) 2: Control (waitlist)	8 weeks, 3 s/wk 30–40 min *Pilates*: main exercises included hundred, roll-up/-down, single leg circle movements, 1–2 × 3–10 reps (10s per rep)	Not reported		Not reported Not reported
				Method: Blood samples before and 48h after intervention (in the morning). Serum analyzed by ELISA kit (BDNF: *Boster Bio, CA, US*). Centrifugation not reported. Storage −80 °C.		
				Acute:	Chronic: BDNF ↑_BG_	
**Joergensen et al., 2019**	RR (Interferon-α or -β) n = 30 (26.7/73.3%) EDSS: 3.0 Age: 44.5 TSD: 7.0	1: RT 2: Control (habitual lifestyle)	24 weeks, 2 s/wk 30 min *RT (whole body)*: 3–5 × 10 reps at 15RM progressing to 6 reps at 6RM	No (primary outcome: total brain volume)		Knee ext + flex EMG ↑_BG_ Knee ext + flex strength ↑_BG_ Brain structure: Total brain volume (↑_BG_) Cortical thickness ↑_BG (4 of 74 subregions)_ T2 lesion volume + count *From Kjolhede* et al. *2018*
				Method: Acute blood samples before and 0, 15, 45, 75, 120 min after one RT session (RT group) or 30 min rest (control group); Chronic blood samples before and after intervention (after overnight fasting at rest in supine position). Plasma analyzed by ELISA kit (BDNF: *MyBioSource, CA, US*). Centrifugation 1200 g for 10 min at 4 °C. Storage −80 °C.		
				Acute: BDNF	Chronic: BDNF	
**Khademosharie et al., 2018**	SP/PP n = 20 (0/100%) EDSS: 3.2 Age: 20–50 TSD: not reported	1: MIX 2: Control (habitual lifestyle)	12 weeks, 3 s/wk (2 AT, 1 RT) *RT (whole body)*: 60–80% of 1RM, 2–4 × 8–14 reps *AT (rhythmic + jogging)*: 15–60 min, 40–55% of HR_reserve_	Not reported		Disability (EDSS) ↓_BG_Body fat % ↓_BG_ Not reported
				Method: Blood samples before and 48 h after intervention (after overnight fasting). Serum analyzed by ELISA kit (BDNF: *Boster Bio, CA, US*; NGF: *Eastibiopharm, CA, US*). Centrifugation not reported. Storage −80 °C.		
				Acute:	Chronic: BDNF(↑_BG_) NGF	
**Mokhtarzade et al., 2018**	RR n = 61 (34.4/65.6%) EDSS: 1.6 Age: 31 TSD: 7.5	1: AT (OW_I) 2: AT (NW_I) 3: Control (OW_C) 4: Control (NW_C)	8 weeks, 3 s/wk *AT (bicycle)*: 42–66 min, 3 × 10 min upper limbs + 3 × 10 min lower limbs, 60–75% peak power	Not reported		VO_2max_ ↑_BG_ Total body mass BMI Brain structure (blood–brain barrier): S100b ↓_BG (NW_I vs control)_ NSE
				Method: Blood samples before and after intervention (after overnight fasting). Serum analyzed by ELISA kit (BDNF, NGF: *R&D Systems, MN, US*; CNTF, PDGF: *IBL International, Germany*). Centrifugation 3000 g for 12 min at 4 °C. Storage −80 °C.		
				Acute:	Chronic: PDGF ↑_BG_ BDNF ↑_BG (NW_I vs control)_ NGF CNTF	
**Naghibzadeh et al., 2019**	RR n = 26 (0/100%) EDSS: 2–4 Age: 33.4 TSD: not reported	1: MIX 2: Control 3: Swedish massage 4: AT + Swedish massage	8 weeks, 3 s/wk *AT (aquatic)*: 30 min, walking and jumping, balance etc., no intensity reported	Not reported		Knee flex strength ↑_BG (all AT groups vs control)_ Knee ext strength ↑_BG (all AT groups vs control)_ Grip strength Not reported
				Method: Blood samples 48 h before and 48 h after intervention. Plasma analyzed by ELISA kit (BDNF: *Boster Bio, CA, US*; NGF, CNTF: *Chongqing Biospes Co, China*). Centrifugation not reported. Storage −80 °C.		
				Acute:	Chronic: BDNF NGF ↑_BG_ CNTF	
**Ozkul et al., 2018**	RR n = 36 MS (22.2/77.8%) EDSS: 1 Age: 33.8 TSD: 4	1: MIX 2: Control 3: Healthy controls (n = 18)	8 weeks, 3 s/wk *AT (treadmill walk)*: 20–60 min, 60–80% HR_max_ *Pilates*: 60 min, multiple exercises, 10–20 reps per exercise	Yes (with suppressors of cytokine signaling proteins)		Postural stability * 6 min walking distance * ↑_BG_ Fatigue severity scale * ↓_BG_ Not reported
				Method: Blood samples before and after intervention (at rest). Serum analyzed by ELISA kit (BDNF: *Shanghai Sunred Biological technology, China*). Centrifugation 3000 g for 10 min. Storage −40 °C.		
				Acute:	Chronic: BDNF *	
**Rezaee et al., 2020**	RR n = 20 (60/40%) EDSS: 2.4 Age: 28.7 TSD: not reported	1: AT 2: Control	6 weeks, 3 s/wk *AT (bicycle)*: 30 min, 60% of VO_2max_	Yes (with tumor necrosis factor alpha)		Not reported Not reported
				Method: Acute/chronic blood samples before (in the morning) and immediately after 1^st^ and 18^th^ AT session. Serum analyzed by ELISA kit (VEGF: *ZellBio GmbH, Germany*). Centrifugation 3000 g for 15 min at 4 °C. Storage −80 °C.		
				Acute: VEGF ↑_BG_	Chronic: VEGF	
**Savsek et al., 2021**	RR n = 28 (82.1/17.9%) EDSS: 2.8 Age: 41 TSD: 11.6	1: AT 2: Control (habitual lifestyle)	12 weeks, 2 s/wk *AT (aerobics)*: 30–40 min (60 min including warm-up and cool-down), 60–70% HR_reserve_	No (primary outcomes: brain structures)		Disability (EDSS) ^#^ Modified fatigue impact scale ^#^ Walking speed ^#^ ↑_BG_ Brain structure: Total brain volume Gray matter brain volume T2 lesion volume + count Cortical lesion volume + count Active lesion volume + count * ↑_BG_ Substructures ↑_BG (5 of 15 substructures)_
				Method: Blood samples before and after intervention (in the morning at rest). Serum analyzed by ELISA kit (BDNF: *R&D Systems, MN, US*). Centrifugation 3500 g for 5 min. Storage −20 °C.		
				Acute:	Chronic: BDNF ^#^ (↑_BG_)	
**Schulz et al., 2004**	RR/SP/PP n = 28 (32/68%) EDSS: 2.3 Age: 39.5 TSD: 11.4	1: AT 2: Control (waitlist)	8 weeks, 2 s/wk *AT (bicycle)*: 30 min, 75% of Watt_max_	Not reported		VO_2max_ Not reported
				Method: Blood samples before and after intervention. Serum analyzed by ELISA kit (BDNF: *Promega, WI, US*; NGF: *sensitive and specific two-site enzyme immunoassay*). Centrifugation not specified. Storage −80 °C.		
				Acute:	Chronic: NGF (↓_BG_) BDNF	
**Wens et al., 2016**	RR n = 22 (36.4/63.6%) EDSS: 2.6 Age: 43 TSD: not reported	1: MIX 2: Control (sedentary) 3: HC (n = 19)	24 weeks, 5 s/2 wk 45–75 min (in total) *AT (bicycle or treadmill)*: 1–3 × 10 min, 12–14 on BORG20 Scale *RT*: 1–4 × 10–15 reps, 12–14 on BORG20 Scale	Yes		Knee flex strength ↑_BG_ Knee ext strength ↑_BG_ Fat mass Total body mass Not reported
				Method: Blood samples before and 48h after intervention (at rest). Serum analyzed by Elisa kit (BDNF: *Meso Scale Discovery, MD, US*). Centrifugation 3000 g for 10 mn. Storage −80 °C.		
				Acute:	Chronic: BDNF ↑_BG_	
**Summary**	n = 473 (n = 366 women) (23/77%) Median EDSS: 2.6 Mean TSD: 9.7 years Mean age: 37.4 years	RT: 1 AT: 5 MIX: 7 Pilates: 1	8–24 weeks 2–5 s/wk RT and AT: low-to-high intensity	No. of studies reporting on neurotrophic factors (and ↑_BG_): Acute: BDNF 1/14 (↑_BG_ in 0/1), VEGF 1/14 (↑_BG_ in 1/1) Chronic: BDNF 12/14 (↑_BG_ in 6/12), IGF-1 1/14 (↑_BG_ in 1/1), NT4 1/14 (↑_BG_ in 1/1), NT3 1/14 (↑_BG_ in 1/1), NT4/5 1/14 (↑_BG_ in 1/1), GDNF 1/14 (↑_BG_ in 0/1), CNTF 3/14 (↑_BG_ in 0/3), NGF 4/14 (↑_BG_ in 1/4), PDGF 1/14 (↑_BG_ in 1/1), VEGF 1/14 (↑_BG_ in 1/1)		No. of studies reporting on physiological/functional outcomes (and ↑_BG_): 11/14 (↑_BG_ in 10/11; one or more outcomes) No. of studies reporting parallel ↑_BG_ (one or more outcomes) in physiological/functional outcomes and acute neurotrophic factors: 1/2 or chronic neurotrophic factors: 8/11 No. of studies reporting parallel ↑_BG_ (one or more outcomes) in neuroprotection and acute neurotrophic factors: 0/1 or chronic neurotrophic factors: 2/3

Arrows denote significant (*p*-value < 0.05) increase ↑ or decrease ↓. Parentheses around arrows denote trends (*p*-value < 0.10). BG denote between-group changes (i.e., group × time interaction). Absence of arrows denote no between- or within-group changes. Gray text denotes reported data from groups that were not included; ^#^ denote data interpretation based upon mean (95% confidence interval); * denote data interpretation based upon median (interquartile range). RR: relapsing–remitting. SP: secondary progressive. PP: primary progressive. EDSS: expanded disability status scale. TSD: time since diagnosis. Min: minutes. AT: aerobic training. RT: resistance training. MIX: mixed training. HR: heart rate. HRmax: maximal heart rate. BDNF: brain-derived neurotrophic factor. IGF-1: insulin growth-like factor 1. Ext: extension. Flex: flexion. NGF: nerve growth factor. GDNF: glial cell-derived neurotrophic factor. CNTF: ciliary neurotrophic factor. PDGF: platelet-derived growth factor. VEGF: vascular endothelial growth factor. S100b: S100 calcium-binding protein B. NSE: neuron-specific enolase. SDMT: symbol digit modalities test. VLMT: verbal learning and memory test. TAP: test battery of attention. OW_I: overweight intervention group. OW_C: overweight control group. NW_I: normal weight intervention group. NW_C: normal weight control group. RM: repetition maximum. VO2max: maximal oxygen uptake.

## Data Availability

No data available as this was a systematic review.

## Supplementary Materials

The following are available online at https://www.mdpi.com/article/10.3390/brainsci11111499/s1, Table S1: Exact Search Strategies.
