# Supplementary material for: Effects of Exercise Training on Neurotrophic Factors and Subsequent Neuroprotection in Persons with Multiple Sclerosis—A Systematic Review and Meta-Analysis
Source: Brain Sci. 2021 Nov 12;11(11):1499. doi: 10.3390/brainsci11111499 (PMC8615767; doi:10.3390/brainsci11111499)
Supplement: Supplementary file 1 [file brainsci-11-01499-s001.zip › brainsci-1449017-supplementary.pdf]

Supplementary Table S1

Exact Search Strategies

Table showing the exact search strategies in five different databases.

Database	Date	Number	Search terms
Pubmed	07.07.21	29	(((("Nerve Growth Factors" OR ("nerve growth factors"[MeSH Terms] OR ("nerve"[All Fields] AND "growth"[All Fields] AND "factors"[All Fields]) OR "nerve growth factors"[All Fields] OR ("nerve"[All Fields] AND "growth"[All Fields] AND "factor"[All Fields]) OR "nerve growth factor"[All Fields] OR "nerve growth factor"[MeSH Terms] OR ("nerve"[All Fields] AND "growth"[All Fields] AND "factor"[All Fields]))) OR ("brain-derived neurotrophic factor"[MeSH Terms] OR ("brain-derived"[All Fields] AND "neurotrophic"[All Fields] AND "factor"[All Fields]) OR "brain-derived neurotrophic factor"[All Fields] OR ("brain"[All Fields] AND "derived"[All Fields] AND "neurotrophic"[All Fields] AND "factor"[All Fields]) OR "brain derived neurotrophic factor"[All Fields])) OR ("brain-derived neurotrophic factor"[MeSH Terms] OR ("brain-derived"[All Fields] AND "neurotrophic"[All Fields] AND "factor"[All Fields]) OR "brain-derived neurotrophic factor"[All Fields] OR "bdnf"[All Fields])) OR neurotrophic[All Fields]) AND ("Multiple Sclerosis"[Mesh] AND ((("Exercise"[Mesh] OR "Physical Therapy Modalities"[Mesh]) OR ("physical therapy modalities"[MeSH Terms] OR ("physical"[All Fields] AND "therapy"[All Fields] AND "modalities"[All Fields]) OR "physical therapy modalities"[All Fields] OR ("physical"[All Fields] AND "therapy"[All Fields]) OR "physical therapy"[All Fields])) OR ("exercise"[MeSH Terms] OR "exercise"[All Fields])))
Embase	07.07.21	119	('exercise'/exp OR (physical AND therapy)) AND (multiple AND sclerosis OR 'multiple sclerosis'/exp) AND (neurotrophic AND factors OR neurotrophins OR (brain AND derived AND neurotrophic AND factor) OR bdnf OR 'neurotrophic factor'/exp OR (nerve AND growth AND factor) OR (neurotrophic AND factor) OR neurotrophic)
Web of Science	07.07.21	107	((“disseminated sclerosis” OR “multiple sclerosis”) AND (exercise OR “physical therapy” OR “physical activity”) AND (“neurotrophic factor” OR “Brain

			Derived Neurotrophic Factor” OR BDNF OR “Nerve Growth Factor” OR neurotrophins))
SportDiscus	07.07.21	8	((multiple sclerosis OR “multiple sclerosis”) AND (“exercise” OR “physical therapy” OR exercise OR physical therapy) AND (neurotrophic OR brain derived neurotrophic factor OR bdnf OR nerve growth factor OR “growth factor” OR neurotrophins))
Scopus	07.07.21	74	(((TITLE-ABS-KEY (exercise)) OR (TITLE-ABS-KEY ("physical activity")) OR (TITLE-ABS-KEY ("physical activity"))) AND (TITLE-ABS-KEY ("multiple sclerosis"))) AND ((TITLE-ABS-KEY ("brain derived neurotrophic factor")) OR (TITLE-ABS-KEY ("neurotrophic factor")) OR (TITLE-ABS-KEY ("neurotrophic factors")) OR (TITLE-ABS-KEY (bdnf)) OR (TITLE-ABS-KEY ("nerve growth factor")))
